# Multifactorial 10-Year Prior Diagnosis Prediction Model of Dementia

**DOI:** 10.3390/ijerph17186674

**Published:** 2020-09-14

**Authors:** Ana Luiza Dallora, Leandro Minku, Emilia Mendes, Mikael Rennemark, Peter Anderberg, Johan Sanmartin Berglund

**Affiliations:** 1 Department of Health, Blekinge Institute of Technology, 371 79 Karlskrona, Sweden; peter.anderberg@bth.se (P.A.); johan.sanmartin.berglund@bth.se (J.S.B.); 2 School of Computer Science, University of Birmingham, Birmingham B15 2TT, UK; l.l.minku@bham.ac.uk; 3 Department of Computer Science, Blekinge Institute of Technology, 371 79 Karlskrona, Sweden; emilia.mendes@bth.se; 4 Faculty of Health and Life Sciences, Linnaeus University, 351 95 Kalmar, Sweden; mikael.rennemark@lnu.se

**Keywords:** dementia, prognosis, modifiable risk factors, decision tree, cost sensitive learning, wrapper feature selection, machine learning

## Abstract

Dementia is a neurodegenerative disorder that affects the older adult population. To date, no cure or treatment to change its course is available. Since changes in the brains of affected individuals could be evidenced as early as 10 years before the onset of symptoms, prognosis research should consider this time frame. This study investigates a broad decision tree multifactorial approach for the prediction of dementia, considering 75 variables regarding demographic, social, lifestyle, medical history, biochemical tests, physical examination, psychological assessment and health instruments. Previous work on dementia prognoses with machine learning did not consider a broad range of factors in a large time frame. The proposed approach investigated predictive factors for dementia and possible prognostic subgroups. This study used data from the ongoing multipurpose Swedish National Study on Aging and Care, consisting of 726 subjects (91 presented dementia diagnosis in 10 years). The proposed approach achieved an AUC of 0.745 and Recall of 0.722 for the 10-year prognosis of dementia. Most of the variables selected by the tree are related to modifiable risk factors; physical strength was important across all ages. Also, there was a lack of variables related to health instruments routinely used for the dementia diagnosis.

## 1. Introduction

### 1.1. Background

Dementia refers to a set of complex neurodegenerative disorders that are characterized by progressive cognitive deterioration and increasing disabilities that affect the individual’s independence, social and professional functioning [1,2]. It affects mostly the older adult population, where early symptoms are characterized by difficulty in remembering recent events, and evolve to more serious ones like disorientation, mood swings, aggravated memory loss, confusion, changes in behavior, impaired gait and difficulty in speaking and swallowing, all of which lead to very poor quality of life [1,3]. Additionally, mortality risks for individuals with dementia are two times higher than for persons without dementia; and they may also have to handle comorbidities that may play a role in accelerating their decline in function [4]. To aggravate this scenario, there is no known cure, and the available symptomatic treatments show limited benefits with regard to cognitive abilities [3]. There is also significant uncertainty surrounding dementia pathology, such as what exactly triggers it and how its progression unfolds. Such uncertainty makes the development of dementia treatments and interventions much more difficult [1]. Another difficult aspect of dementia disorder is that it brings negative consequences to caregivers (usually next of kin), whose burden cannot be overlooked [5]. These persons deal with this complex condition for years and report low confidence in managing it, in addition to being prone to negative health outcomes themselves, like high levels of strain, depressive symptoms, risk of alcohol-related problems, etc. [1,5]. The families of individuals affected by dementia also suffer considerable financial impact due to the costs of care and reduction in income [6]. In the public health sphere, dementia represents a significant cost which is expected to increase in the coming decades. In 2015, the global sum of direct medical costs and social and informal care for dementia was estimated to be US$818 billion, which amounted to 1.1% of global gross domestic product [6]. Since old age is believed to be the biggest risk factor for dementia, and life expectancy is growing worldwide, the global costs for dementia are estimated to rise to US$2 trillion by the year 2030. This can place a huge strain on healthcare systems around the world [6]. With regard to the health economics of dementia, nowadays, the main costs associated with this disorder are directed towards compensating for the decline in an individual’s ability to function, instead of treatment or efforts towards prevention [1]. 

It is worth noting that dementia disorder has several subtypes, with the most common being Alzheimer’s disease, Vascular dementia, dementia with Lewy Bodies and Frontotemporal dementia [7]. However, mixed pathologies are not uncommon, especially with regard to Alzheimer’s disease in conjunction with Vascular or Lewy Bodies dementia. Also, uncommon subtypes tend to be misdiagnosed as Alzheimer’s disease [7]. The work presented herein makes no distinction between subtypes, and the term ‘dementia’ is used to refer to all forms.

### 1.2. Addressing the Dementia Epidemic

Prevention and risk reduction have been identified by the World Health Organization as key elements to focus on to decrease the worldwide impact of the dementia epidemic [1,6]. In this sense, the identification of modifiable risks is imperative, and prognostic estimates become more and more important. Prognostic estimates usually refer to the expected outcome of a disease; however, a prognosis can also be employed to predict future outcomes of healthy individuals [8]. Prognostic estimates are useful to identify patterns of disease progression, to support public entities in creating and maintaining healthcare programs to address the epidemic, and to aid patients and their families in understanding their condition in order to participate in shared decisions with health providers [8].

However, any research investigating a disorder as complex as dementia is difficult, especially with regard to traditional trials, as they suffer from major obstacles. For instance, certain health conditions that have high prevalence among older persons (e.g., cardiovascular disease) cannot be left untreated in a control group; blinding is a challenge in trials related to lifestyle interventions; and since dementia is a multifactorial disorder, the assumption of one-dimensionality may hinder the results [1,9]. Another significant challenge in such research is that evidence shows that changes in the brains of individuals who develop dementia can start to occur as early as 10 to 15 years before diagnosis; therefore, preventive interventions should take this into account, despite the risk that such considerations could make research efforts more costly [10]. Due to the aforementioned challenges, research on the risks and prevention of dementia could benefit from data-centered approaches such as machine learning.

### 1.3. Machine Learning Approaches for the Prognosis of Dementia

Machine learning is a field of the artificial intelligence discipline which aims to design algorithms that are able to learn from data. The reasons for employing such algorithms are related to the limited human capacity to deal with large amounts of data, multiple variables and changing environments, i.e., settings which would hinder, if not impede, manual coding or analysis [11]. These involve tasks that are too complex to be defined (except for examples of inputs and outputs), the extraction of meaningful correlations in large amounts of data, and works in unknown or changing environments [12]. The knowledge acquired during the machine learning process is encapsulated in the form of a model, which, when faced with new data, is able to perform the designated task [12].

When applied to a prognostic setting, machine learning algorithms are able to analyze the health trajectories of a large number of individuals to identify patterns of progression that lead to specific health outcomes of interest [13]. Being able to work with large amounts of multifactorial data, machine learning technology could be beneficial in the investigation of prognostic estimates for dementia. However, after a systematic literature review that explored machine learning approaches for prognosis [14], it was concluded that, based upon 37 primary studies, the research has been very focused on neuroimaging for identifying and validating biomarkers in the brain to predict the development of dementia from its prodromal stage, i.e., mild cognitive impairment (MCI), and usually in a time frame of 3 years [14]. These studies scarcely considered modifiable risk factors.

One of the most significant initiatives in this direction is the Alzheimer’s Disease Neuroimaging Initiative (ADNI), which aims to identify biomarkers to be used in treatment trials and pharmaceutical research [15]. Research related to dementia treatments is important not only in the search for a cure, but also to develop drugs aimed to delay the progression and to shorten the time spent in the most severe stages of this disorder, characterized by the lowest quality of life for the patients and families, and increased costs for healthcare systems [1]. Note, however, that the aforementioned research focuses on persons who are already at a major risk of developing dementia, since reported conversion rates from MCI to dementia can be as high as 40% [16,17,18]. Their focus on the use of medical images has directly influenced the choice of the machine learning techniques that were employed; these were, in the vast majority of cases, Support Vector Machines, followed by Neural Networks approaches [14]. These are black-box methods which do not support the interpretation of the resulting model regarding how the predictions were made.

Only one study identified in the systematic literature review considered both modifiable and nonmodifiable risk factors. However, the study sample consisted of already cognitive impaired subjects, and hence, individuals who were already at major risk of developing Alzheimer’s Disease, and the prediction was given in a short interval, i.e., 5 years [19]. 

The machine learning technique that was employed in this study was decision trees. The decision trees technique creates a model for representing knowledge in the form of IF-THEN rules that are intuitive and highly interpretable, which is the reason why it is widely used in medical research for identifying prognostic subgroups [20]. For the specific case of dementia, the interpretability feature of the decision trees technique could be very beneficial, not only for identifying risk factors, but also for pointing out their possible interactions, which can contribute to a better understanding of this disorder. Since not much is known about how dementia unfolds, a “black box” model for early detection may not yield significant benefits to patients; furthermore, it also raises ethical issues related to the early detection of a highly debilitating condition without cure and without providing means for symptom relieving therapy. Such ethical concerns should be highly regarded in the case of dementia, as evidence shows that its early diagnosis is related to a risk of depression, suicide and requests for euthanasia [21].

In summary, dementia research faces challenges regarding traditional research and would benefit from data centered machine learning approaches, which are able to provide predictions that consider various factors, in a time frame large enough for the application of interventions that could possibly delay or prevent the onset of the disease. What is evidenced in the literature regarding machine learning for dementia prediction is studies that make their predictions over smaller time frames (usually 3 to 5 years), and which are based almost exclusively on neuroimaging. 

In the present study, a multifactorial approach for the 10-year prediction of dementia is proposed. It is important to note that, while a 15-year prediction would be a better time frame for a multifactorial prediction tool, due to limitations in the data, it was not possible to approach the problem in this way. The 10-year time frame was considered in the present study as being a long-term prediction, which could allow interventions to be implemented for the prevention or delay of the onset of dementia. This could be considered a differential to the present study, since the aforementioned studies in the literature which approach the problem over smaller time frames (3 and 5 years) explicitly consider subjects already at the prodromal stage of dementia disorder (MCI), whereas in the approach proposed herein, there is a higher chance of studying the prediction of dementia in its preclinical stages. This is important, since the approach constitutes a prognostic and not a diagnostic model.

### 1.4. Study’s Aim

Given the complexities and current status of dementia research, this study proposes a machine learning approach, based on decision trees, aiming at the prediction of dementia in 10 years. The proposed approach addresses research gaps in the following ways:

- A longitudinal approach that investigates the prediction of dementia in a cohort of older individuals who did not present a diagnosis of dementia at baseline (2000 to 2003) and their development (or not) of dementia at the 10-year mark of the study (2010 to 2013). This could provide a time frame large enough for the application of interventions for delaying or preventing the onset of dementia.
- A broad multifactorial approach that considers 75 variables related to modifiable and nonmodifiable risk factors in the following categories: demographic, social, lifestyle, medical history, biochemical tests, physical examination, psychological assessment and multiple health instruments relevant to the dementia evaluation. This approach considers modifiable and nonmodifiable factors which cover many domains in which interventions could be investigated.
- An interpretable approach, which employs decision trees, in order to identify possible risk factors and prognostic subgroups of interest. The decision tree approach not only identifies factors which are important for the prediction process, but examines how they interact with each other, which could lead to possible prognostic subgroups.

## 2. Materials and Methods

### 2.1. Population

The sample data used herein is a subset from the Swedish National Study on Aging and Care (SNAC). The SNAC encompasses a longitudinal cohort that has been collecting multifactorial data from the older adult population in Sweden, which aims at “building reliable, comparable, longitudinal data bases, which will constitute an effective infrastructure for research on aging and care provision to the elderly” [22]. The SNAC project was designed as a multipurpose project to study the aging population’s health, as well as the provided social care, and contains a database (more information on the data and examples on surveys can be found at https://www.snac-k.se/for-researchers) with data regarding physical examination, psychological assessment, social factors, lifestyle factors, medical history, etc. The SNAC project started in 2000 and is ongoing. The SNAC cohort contains a subset of the aging population in Sweden that collects data of individuals aged 60 years or more into defined age groups (60, 66, 72, 78, 81, 84, 87, 90, 93 and 96+ years) [22]. The SNAC data is collected from four sites, which represent two Swedish counties, one borough and one municipality; they are, respectively, Skåne, Blekinge, Kungsholmen and Nordanstig. Such a range aims to gather representative national data from urban (Kungsholmen), rural (Nordanstig) and mixed areas (midsized cities with rural areas) (Skåne, Blekinge) in order to provide an average representation of the elderly population in Sweden.

The population used herein was the baseline examination of the SNAC-Blekinge, with data ranging from 2000 to 2003. Although it is evidenced in the literature that environmental factors may play a role in dementia occurrence [23,24], this study is based upon generalized factors, and differences between urban and rural areas are not included. The recruited subjects underwent extensive examinations and interviews by physicians, nurses and psychologists [22]. In cases of disabilities, an examination team did the examinations and interviews at the subject’s home, and proxy interviews were conducted with relatives of the subjects whenever necessary, and with the subject’s consent [22].

The following criteria were used to determine the exclusion of subjects in this study: (i) subjects who already had dementia at baseline; (ii) subjects who had missing values at the outcome variable (dementia diagnosis); (iii) subjects who presented more than 10% of missing values in the input variables; (iv) subjects who passed away before the 10-year study mark; and (v) subjects who were diagnosed with dementia before the 10-year mark, as they could already have advanced dementia at baseline, which could bias the early prediction of dementia.

The SNAC Blekinge baseline consisted of 1402 subjects. After applying the aforementioned exclusion criteria, the study sample consisted of 726 subjects (313 males and 413 females), among which 91 (12.5%) were given a diagnosis of dementia at the 10-year mark and 635 were not. The demographics of the study sample are shown in Table 1.

### 2.2. Ethics and Data Privacy

This study was carried out in accordance with the Declaration of Helsinki and was approved by the Research Ethics Committee at Lund (LU 604-00, LU 744-00). Written informed consent was collected from all subjects. All data were anonymized and stratified by age and gender.

### 2.3. Outcome Variable: Diagnosis of Dementia at the Snac 10-Year Mark

The outcome variable that the decision tree aims to estimate is the dementia diagnosis given by physicians 10 years from the SNAC baseline. The given diagnosis of dementia followed the guidelines of the International Statistical Classification of Diseases and Related Health Problems-10th Revision (ICD-10) [25] and the Diagnostic and Statistical Manual of Mental Disorders (DSM-5) [26]. No distinctions between subtypes of dementia were made, since mixed pathologies are common, and uncommon subtypes tend to be misdiagnosed by Alzheimer’s disease [7].

### 2.4. Input Variables

The input variables used to build the decision tree prognostic estimate models were selected from the SNAC database by senior researchers specialized in geriatrics and gerontology (authors 4 and 6) who adopted a broad approach in order to consider variables of diverse types that could influence the onset of dementia. This selection was based on the evidence in the literature, which suggests the influence of the selected variables in the dementia disorder [24,27,28]. It is also worth noting that all of the variables chosen for the SNAC project were chosen based on evidence of being important in the aging process (health/disease, social and support network, lifestyle factors, material conditions and personal resources), in addition to data regarding the use of care services [22]. In total, 75 variables were selected, which encompassed the following categories: demographic, social, lifestyle, medical history, blood test, physical examination, psychological and the assessment of multiple health instruments relevant to the dementia evaluation at the study baseline (2000–2003). Table 2 shows a list of the selected variables. Descriptions, possible values and number of missing values of all input variables are shown in Supplementary Material 1.

### 2.5. Data Preparation

The K-Nearest Neighbors (KNN) multiple imputation approach was employed to handle missing data [39]. This technique works by finding the K entries of data that are most similar (near) to an entry that contains missing data. Then, the KNN imputation fills its missing values with the mean (in the case of numeric variables) or the most frequent value of the K most similar neighbors (in the case of categorical variables) [39].

In the present study, the KNN imputation was applied separately for entries of the majority class (no dementia at 10 years mark) and minority class (dementia at 10 years mark). This was done because the sample used to build the prognostic estimates presented a pronounced class imbalance (12.5% on the minority class to 87.5% on the majority class), so the risk of contaminating the minority class with data from the majority class was mitigated. This is in line with the literature on missing values on binary response decision trees, which has been shown to provide better classification performance when imputation is done separately [40].

The number of nearest neighbors used in the KNN imputations was set to K = 3. This choice was based on literature findings that argue about limiting the number of K to avoid distortion of the original variability of the data; one such example is the critical evaluation of Nearest Neighbors Imputation on medical datasets by Beretta and Santaniello that recommends K = 3 [41].

### 2.6. Decision Tree Approach

A decision tree approach was applied to predict dementia at the 10-year mark of the study. This prediction is given by automatically building a binary classifier based on existing data by using machine learning. The classifier defines subjects as having ‘no dementia’ (negative class) or ‘dementia’ (positive class), as specified by the outcome variable.

The aim of the decision tree algorithm is to partition the available data into groups (nodes) which are as homogenous as possible. Homogeneity in this context means that these groups minimize intravariability and maximize intervariability, i.e., they contain a larger proportion of one class than the others. The decision tree algorithm used herein was the Classification and Regression trees (CART) algorithm, which performs binary recursive partitioning [42]. The CART approach tests each input variable to its capacity of splitting the data points with regard to an outcome variable, creating ‘left’ and ‘right’ nodes. This process repeats recursively until no more splits are possible [42]. The nodes created by the last splits in the tree are called terminal nodes and provide the class prediction.

The splits in the CART algorithm that was used to build the models in this paper are based on the impurity split criteria given by the Gini index [42], which, for node *t* and input variable *x*, is calculated as follows [43]:

(1) $$Gini\left(t\right)=1-\overset{c}{\underset{i=1}{\sum }}P_{i}^{2}$$

where *c* is the number of classes, which in our case is two; and *P_i_* is the proportion of the class *i* in the node *t*. The Gini calculation for the ‘left’ and ‘right’ nodes are then given, respectively, as follows:

(2) $$Gini\left(t_{left}\right)=1-\;\overset{c}{\underset{i=1}{\sum }}P_{i|left}^{2}$$

(3) $$Gini\left(t_{right}\right)=1-\;\overset{c}{\underset{i=1}{\sum }}P_{i|right}^{2}$$

where *P_i|left_* and *P_i|right_* refer to the proportion of the class *i* in the left and right nodes, respectively. The goodness *g* of the given split s of the node *t* is calculated as follows:

(4) $$g\left(s,x,t\right)=Gini\left(t\right)-\;P_{left}\;Gini\left(t_{left}\right)-\;P_{right}\;Gini\left(t_{right}\right)\;$$

The goodness of split will be calculated for the input variables in the dataset. In order to find the best split of a given node, the CART algorithm will search for the input variable which will provide the maximum goodness of split [43].

### 2.7. Model Building

For the model building, two common problems in studies with medical databases needed to be addressed: high class imbalance (12.5% on the minority class and 87.5% on the majority class); and high dimensionality (high number of input variables in relation to the number of entries). Both are known to hinder the performance of decision tree algorithms [44,45].

Addressing these problems is an active topic of research in the computer sciences, where the most explored approaches are: resampling, which modifies the distribution of the data used for training the predictive models toward balancing the classes; cost-sensitive learning, which imposes a penalty for the misclassification of the most important class; one-class learning, which is an unsupervised approach that learns by imposing a similarity threshold for the classification; and feature selection, which selects the set of features that enables the classifier to achieve optimal performance [46,47]. A systematic comparison study by Wasikowski and Chen [47] investigated resampling and feature selection approaches for high-dimensional imbalanced problems, achieving better performance with the use of feature selection, but no performance improvement with resampling techniques. He and Garcia [48] conducted a review of state-of-the-art techniques for imbalanced learning and found that in many empirical domains, cost-sensitive learning approaches achieved better results than resampling.

Based on such findings, and on the supervised nature of the problem at hand, the high-dimensionality and class imbalance were addressed herein by a cost-sensitive approach and a wrapper feature selection method. These are detailed below.

#### 2.7.1. Cost-Sensitive Learning

The consequence of having an imbalanced dataset is that the trained classifier tends to be biased towards the majority class, having very poor ability to recognize examples of the minority class. The cost-sensitive learning approach works by applying a heavier penalty on misclassifying the minority class. In so doing, it attributes higher weights to the minority class, thus addressing the bias.

The approach used herein was instance weighting based on class distribution, in a way that the weights for each of the classes are inversely proportional to the number of class instances in the training data [48,49]. The weights in the decision tree algorithm are used in the Gini index criterion for finding splits, and also on the terminal nodes in the class prediction, which is given by the weighted majority vote [50].

#### 2.7.2. Wrapper Feature Selection

To address the high number of input variables in relation to the number of entries, the Recursive Feature Elimination (RFE) feature selection method was employed in order to select the most important variables for the classification. This wrapper method assesses multiple different models composed of different combinations of input variables in order to find the optimal subset of variables to maximize a performance metric of choice [44].

### 2.8. Experimental Setup

**Figure 1:**
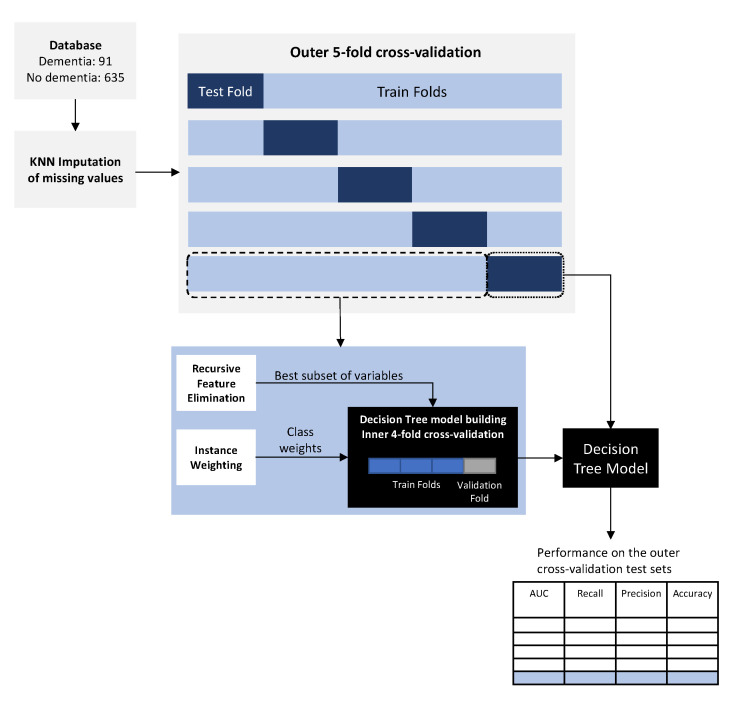
Overview of the proposed approach.

Class weighting and RFE were performed before training the decision tree models. An overview of the proposed approach is shown in Figure 1.

All experiments were performed using a stratified nested cross-validation setup. This approach performs outer and inner cross-validations in such a way that in each iteration, one-fold of the outer cross-validation is used for testing, and the remaining for the inner cross-validation, which is responsible for hyperparameter tuning (see Figure 1) [51,52]. Using the nested approach for cross-validation produces a more reliable estimate of error, since data being used to estimate the model’s performance is not being used for optimization. This avoids the high risk of producing overly-optimistic results, which occurs when using only one test set, as is done in more traditional machine learning experimental setups [51,52]. After performing nested cross-validation and reporting the values of the evaluation metrics for each of the outer cross-validation test sets, we select the model which produces the median performance results of the outer cross-validation test sets for further analysis. The stratified part of the proposed approach means that for all the folds of both inner and outer cross-validations, the proportions of both the minority and majority classes remain the same as in the original sample. Due to class imbalance, the experiments were conducted in a 5-fold outer, 4-fold inner nested cross-validation setup in order to have enough examples of the minority class in each fold.

For the specific decision tree algorithm implementation in R (version 3.4.1) used in this study, we refer to the book by Kuhn and Johnson [44]. The R package employed for training the decision tree and feature selection was the caret, and imputation was performed with the VIM package.

### 2.9. Evaluation Metrics

The main metric that drove the evaluation of the models was the Area Under the Curve (AUC), due to it being undisturbed by skewed class distributions [53]. Other metrics considered for assessing the performance in the experiments were Accuracy, Recall and Precision [54].

## 3. Results

The performance metrics obtained in each of the outer cross-validation test sets of the nested stratified cross-validation procedure are shown in Table 3.

General lower values for the Precision metric were a consequence of the class weighting approach employed to deal with the significant class imbalance of the used sample. This makes the misclassification of the positive class costlier than the inverse, minimizing the gravest error. In a diagnostic tool, this would be a poor scenario, but in the present study, the aim is to conduct a prognostic analysis on the factors that could influence the development of dementia 10 years later, so this type of error has a reduced importance.

**Figure 2:**
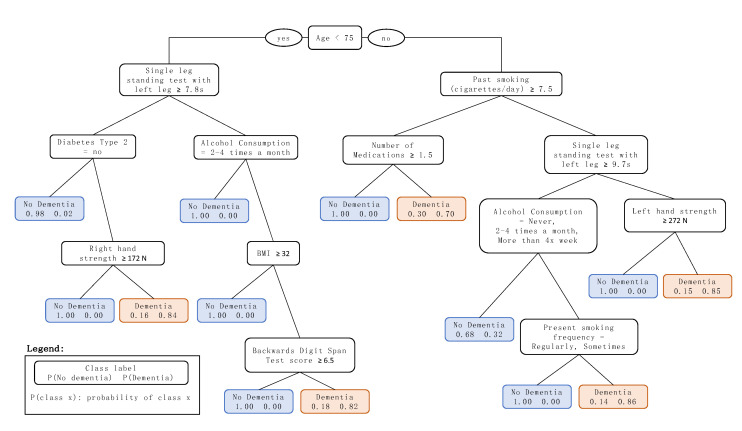
Resulting decision tree model for the prediction of dementia in 10 years. Throughout the decision tree, the left branches refer to ‘yes’ with regard to the rule of the node, and the right branches refer to ‘no’.

The median model, which presents the median AUC, was model 2 with an AUC of 0.735, Accuracy of 0.745, Recall of 0.722 and Precision of 0.289. The decision tree that visually represents this model is shown in Figure 2. A description of the variables with possible values selected by the final tree is shown in Table 4.

## 4. Discussion

### 4.1. Discussion of the Results

Age was chosen by the algorithm as the decision tree root node (see Figure 2), at a threshold of 75 years, thus indicating the importance of such a classification. This is in line with the literature that attributes age as the major risk factor for dementia, pointing out increased risk for the ages greater than 65 years, which accounts for 95% of the cases [1]. The threshold of 75 years, chosen by the decision tree model shown in Figure 2, could be attributed to the fact that the study sample comprises subjects older than 60 years (at the baseline), excluding subjects on the early onset who developed dementia before the 10-year mark of the study. Using the threshold of 75 years, the following sections will detail the decision tree classification for branches of subjects older and younger than 75 years.

#### 4.1.1. Prediction of Dementia for Subjects 75 Years and Older at Baseline

The most striking factor of the branch of subjects 75 years and older is the presence of two variables related to smoking, which not only relate to whether an individual is a present smoker at baseline, but also if they smoked in the past. Smoking is a known risk factor for dementia, and literature findings point out that smoking in midlife and late-life increases the risk of developing dementia (compared with nonsmokers) [56], and may also be related to an accelerated cognitive decline (assessed by the Mini-Mental State Examination [35]) [57].

The variable associated with the number of medications taken regularly by the subjects is related to that of past heavy smokers, and could be interpreted as an indicator of comorbidities (possibly connected to the heavy smoking itself). The presence of comorbidities may be associated with an accelerated decline in function in demented individuals [2]. However, the decision tree points out that individuals who, on average, took less regular medication were associated with dementia 10 years later, which might indicate individuals prone to not seeking medical care, and therefore with a higher chance of presenting undiagnosed conditions.

The inclusion of the hand strength (grip test) and single-leg balancing exams testify to the fact that physical strength is protective against dementia. A poor value on these variables could be related to frailty or sedentary life, which is known to be a risk factor of dementia [1].

The last variable on the branch related to subjects 75 years and older is alcohol consumption frequency. Evidence about the protective benefits of alcohol consumption are not very consolidated in the literature, as no randomized clinical trials have been performed on the subject, but observational studies suggest that moderate consumption seems to be protective against cardiovascular disease (a known risk factor of dementia [1]), while abstinence or heavy drinking seem to be a factor of risk [58]. This is in line with the thresholds chosen by the decision tree.

#### 4.1.2. Prediction of Dementia for Subjects Younger than 75 Years at Baseline

The variables in the branch related to the subjects younger than 75 years are somewhat similar to those of older subjects in relation to hand strength (grip test), single-leg balancing and alcohol consumption frequency.

Another important variable in this branch is the subject’s medical history with regard to diabetes Type 2. The risk of dementia in individuals with this condition is reported to be higher than in individuals without it [59], and there is a theory being actively researched which considers Alzheimer’s Disease as a diabetes Type 3, as it seems that there are common molecular and cellular features in diabetes Types 1 and 2 which are associated with cognitive decline in the older adult population [60]. It is also interesting to note that in the branch of individuals with diabetes Type 2, the following node that relates to hand strength has a threshold which is much lower compared to the branches of subjects 75 years or older, with a difference of 100 Newtons. This might be a direct consequence of the weakening effects of diabetes Type 2.

A BMI threshold of 32 kg/m^2^ was also selected as a split in the tree, which is commonly referred to as the threshold for being considered overweight. The relationship between BMI and dementia, and it being a risk factor or not, is not consolidated in the literature, and findings thereof are conflicting; trials on the older adult population showed that higher BMI values are both protective and a risk factor [61,62]. In the present study, lower BMI values in older individuals were predictive factors of dementia in a 10-year time frame.

The only health instrument that was selected as a predictive factor of dementia was the Backwards Digit Span test. This is a neuropsychological instrument that is used to assess the working memory [63]. Since mild memory loss is one of the earliest symptoms of dementia, a poor result in this test might indicate the start of the development of the disorder, but no literature was found to relate scores in Backward Digit Span test and the risk of development of dementia.

### 4.2. Related Work

This study proposed a decision tree approach for the 10-year prediction of dementia, which achieved an AUC of 0.745 and Recall of 0.722. The proposed approach investigated multiple domains in order to derive prognostic estimates, assessing 75 variables related to demographic, social, lifestyle, medical history, biochemical tests, physical examination, psychological assessment and diverse health instruments relevant to the dementia disorder. The approach investigated herein not only identified predictive factors (and thresholds for these factors) related to the long-term prediction of dementia, but also possible interactions between these, which is enabled by the structure provided by the decision tree model. These interactions could be further investigated in clinical trials as being potential prognostic subgroups of importance for dementia.

The main findings of our assessment are the following: (i) even though both modifiable and nonmodifiable factors were considered for the prediction, the majority of the variables selected by the decision tree were related to modifiable factors; (ii) poor physical strength was an important predictive factor of dementia across all ages of the study sample; and (iii) the decision tree selected almost no variables related to health instruments that are used nowadays in the assessment of dementia (e.g., Mini-Mental State Examination [35], Clock Drawing Test [36]).

With the exception of age, past smoking, number of medications and the Backwards Digit Span test, the remaining variables present in the prognostic tree can be considered modifiable factors. These are related to physical strength, present smoking, BMI, diabetes Type 2 and alcohol consumption. An important consideration about the number of medications variable is that, although in this study it was characterized as a nonmodifiable factor, there are cases in which it is possible for clinicians to alter the medications taken regularly by a patient, for reasons such as when the burden outweighs the benefits from the drug in use, worsening of the condition, risk of dangerous interactions with other medications, or even the diagnosis of a new condition that needs pharmacological therapy. These results are promising, as 10 years could be a considerable time frame for the implementation of interventions with regard to these factors for delaying or even preventing dementia, which could mean major individual, societal and financial benefits. However, without further investigation trials on these specific factors, it is not possible to assert that the proposed factors are in fact protective against dementia.

Randomized clinical trials on multidomain interventions related to modifiable risks to investigate the benefits to cognition or dementia incidence in the older population were conducted, the most notable being the Finnish Geriatric Intervention Study to Prevent Cognitive Impairment and Disability (FINGER). The FINGER trial aimed at investigating the effects on cognition of a 2-year lifestyle multidomain intervention that encompassed nutritional guidance, exercise, cognitive training, social activity, and the management of metabolic and vascular risk factors [64]. The population of the study was composed of 1200 individuals (60 to 77 years) already at risk of cognitive decline. The FINGER results showed that the individuals in the intervention group had a positive effect in cognition (score in the Neuropsychological Test Battery [65]), even when correcting for sociodemographic, socioeconomic, cognitive, or cardiovascular factors at baseline [66]. The risk factors identified by the proposed decision tree model in this study are in line with the successful approach of the FINGER RCT, except for the nutritional guidance, that was not considered in our approach, and social factors, which were not selected by the decision tree model. The remaining interventions can be compared:

- Exercise: the FINGER RCT proposed interventions that addressed strength training, aerobics and balance. These can be related to the right-hand strength, left hand strength and single leg standing tests with left leg factors identified by the decision tree model, which regard the hand grip test as a measure of physical strength and the single leg standing test as a measure of balance.
- Metabolic and vascular risk: the FINGER RCT performed anthropometric measurements (weight, blood pressure, hip and waist circumference) every three months for the subjects in the intervention group. The decision tree model identified the modifiable factors for dementia: a medical history of diabetes Type 2 at least 10 years’ prior the dementia diagnosis; past smoking habit (cigarettes/day) and present smoking frequency, which indicates both the present and past habit of smoking; alcohol consumption, which takes into account alcohol consumption habits and BMI, which could be an indicator of obesity. All of these factors are related to metabolic and vascular risks.
- Cognitive training: The FINGER RCT proposed a computer-based intervention that addressed episodic memory, executive function, mental speed and working memory. The decision tree model of the present study identified the Backwards Digit Span Test score, which assesses the working memory of individuals.

Being a 2-year intervention, the FINGER trial brought positive results with regard to the cognition of the participants, with many factors comparable to the present study, which proposed a 10-year prediction tool for dementia. This strengthens the evidence of the merits of the results presented herein and suggests that the identified factors could be strong indicators to be studied in longer trials for the prevention or delay of the onset of dementia.

Another important aspect to be discussed is that among the sub-branches of the tree that leads to dementia in 10 years from the baseline, in all of them but one, there was the presence of a node indicating poor physical strength. Exercise brings a series of health benefits which might act in favor of dementia prevention, e.g., by helping to maintain a healthy weight, reducing diabetes risk, improving cardiovascular function, decreasing glutamine and enhancing hippocampal neurogenesis [67]. Such benefits are believed to reduce neuropathological damage and increase or maintain cognitive reserve [67]. Evidence in the literature of the effects of exercise on lowering the risk of dementia shows favorable results in longitudinal observational studies of over 20 years of follow up [68,69,70,71]. Nevertheless, a recent systematic literature review on physical activity interventions for slowing and delaying cognitive decline, cognitive impairment and dementia in individuals without diagnosed cognitive impairments found insufficient evidence for such claims [72]. However, the identified trials were characterized by single-component interventions in short follow ups (mostly 6 months and a maximum of 2 years) [72]. This might indicate that future trials would benefit from considering longer follow up periods to determine the benefits of exercise, also in conjunction with other modifiable risk factors like the ones identified in the present paper.

Additionally, the lack of variables related to health instruments that are routinely used for assessments of dementia indicates that these instruments may not be sensitive enough for long-term predictions, and may also be insensitive to mild cases that are possibly at the beginning of their development. We can argue that such instruments were conceived for diagnosis and not prognosis, i.e., they are sensitive to early signs that indicate cognitive decline, but are not suited to making long-term predictions. The only health instrument identified by the decision tree was the Backwards Digit Span test, which assesses the working memory of an individual and may have potential for use in detecting early memory loss, one of the early symptoms of dementia. This is important, as most cases of dementia are diagnosed when the disorder is at an already advanced stage [6].

Lastly, considering the results, especially in relation to the modifiable factors, they are all already recommended in some form to prevent chronic illnesses and to maintain a healthy lifestyle. This could also refer to theories that state that dementia is not an unavoidable consequence of aging, and measures to delay and prevent its onset should be investigated [1].

### 4.3. Limitations

The low value for the precision metric, which was a direct consequence of employing the cost-sensitive learning technique, could be seen as a limitation of the proposed method. This feature would be undesirable if our objective were to propose a diagnostic tool; however, the main goal of the proposed approach was to identify factors for the long-term prediction of dementia, minimizing the misclassification of demented cases, which are the most important class to be classified. This was achieved with reasonable success.

It is important to note that commonly used dementia research biochemical laboratory tests related to glucose, urinalysis, cerobrospinal fluid, thyroid, amyloid beta-tau and oxidative stress factors were not available in the SNAC Blekinge database; this could be seen as a study limitation. However, the present study employs a broad range of factors and selected variables which are able to relate to these.

Internal validity threats related to selection threats were mitigated, as the data from the SNAC study consisted of a randomized sample of the aged population in Sweden. With regard to the external validity, it could be argued that the number of subjects was too low to be generalizable. However, the stratified, nested cross-validation method was employed to avoid the selection of an overfitted or overoptimistic model. Also, it is important to note that the results presented herein relate to a Swedish urban sample, and may not be generalizable to other populations with different overall socioeconomic statuses; therefore, further investigation is needed in order to establish the effect of this factor on the results presented herein. Construct validity threats in the present study relate to the question of whether the considered measures represent what the study intended to investigate. The SNAC data used in this study were collected by trained physicians, nurses and psychologists who followed the Good Clinical Practice standards for clinical studies (GDP). Nonetheless, a possible construct threat could arise from the use of 8 variables which had not previously been validated in studies (Descriptions 1 to 8 in the Supplementary Material 1); however, none of these were chosen in the final model. Finally, the conclusion validity threats involve the risk of drawing inaccurate conclusions from the experiments. This threat was mitigated by the use of nested cross-validation and the approach for choosing the final model, which prevented the choice of an overly-optimistic model.

## 5. Conclusions and Future Work

This paper proposed a decision tree model for the prediction of dementia using data from a longitudinal, population-based study (SNAC-Blekinge). Our broad, multifactorial approach investigated social, lifestyle, medical history, blood examination, physical and psychological factors, and diverse health instruments that are currently in use for the assessment of dementia. The model achieved an AUC of 0.745 and Recall of 0.722, and identified diverse modifiable factors that could potentially give rise to interventions attempting a delay or prevent the onset of dementia. 

Future work efforts should be directed towards the investigation of gender-specific prognoses of dementia, especially with the imbalance shown in the incidence between male and female subjects at the 10-year mark. In addition, as this study is based on a single site of the SNAC study (Blekinge), further studies should be undertaken to validate the findings of this study in the other SNAC sites.

## Figures and Tables

**Table 1:** Demographics of the study sample.

		Age at Baseline								
Diagnosis	Gender	60	66	72	78	81	84	87	90+	Total
No dementia at 10 years mark	Male	81	72	47	36	27	19	2	0	284
	Female	81	92	67	38	35	27	7	4	351
Dementia at 10 years mark	Male	1	3	3	5	8	7	2	0	29
	Female	1	3	7	12	11	15	12	1	62
	Total	164	170	124	91	81	68	23	5	726

**Table 2:** List of input variables that were selected by dementia specialists.

Variable Type	Variables
Demographic	Age, Gender,
Social	Education, Holds a Religious Belief or not, Participation in Religious Activities, Voluntary Association, Social Network, Support Network, Loneliness
Lifestyle	Light Exercise, Alcohol Consumption, Alcohol Quantity, Working State at 65 years, Physical Workload, Present Smoker, Past Smoker, Number of Cigarettes a Day, Social Activities, Physically Demanding Activities, Leisure Activities
Medical History	Number of Medications, Family History of Importance, Myocardial Infarction, Arrhythmia, Heart Failure, Stroke, TIA/RIND, Diabetes Type 1, Diabetes Type 2, Thyroid Disease, Cancer, Epilepsy, Atrial Fibrillation, Cardiovascular Ischemia, Parkinson’s Disease, Depression, Other Psychiatric Diseases, Snoring, Sleep Apnea, Hip Fracture, Head Trauma, Developmental Disabilities, High Blood Pressure
Biochemical Test	Hemoglobin Analysis, C-Reactive Protein Analysis
Physical Examination	Body Mass Index (BMI), Pain in the last 4 weeks, Heart Rate Sitting, Heart Rate Lying, Blood Pressure on the Right Arm, Hand Strength in Right Arm in a 10s Interval, Hand Strength in Left Arm in a 10s Interval, Feeling of Safety from Rising from a Chair, Assessment of Rising from a Chair, Single-Leg Standing with Right Leg, Single Leg Standing with Left Leg, Dental Prosthesis, Number of Teeth
Psychological	Memory Loss, Memory Decline, Memory Decline 2, Abstract Thinking, Personality Change, Sense of Identity
Health Instruments	Sense of Coherence [29], Digit Span Test [30], Backwards Digit Span Test [30], Livingston Index [31], EQ5D Test [32], Activities of Daily Living [33], Instrumental Activities of Daily Living [34], Mini-Mental State Examination [35], Clock Drawing Test [36], Mental Composite Score of the SF-12 Health Survey [37], Physical Composite Score of the SF-12 Health Survey [37], Comprehensive Psychopathological Rating Scale [38]

**Table 3:** Performance results in each of the 5-outer cross-validation test sets.

Test Set	AUC	Accuracy	Recall	Precision
1	0.718	0.664	0.790	0.250
**2 (median)**	**0.735**	**0.745**	**0.722**	**0.289**
3	0.827	0.738	0.944	0.315
4	0.763	0.752	0.778	0.304
5	0.712	0.662	0.778	0.237

The test set with the median results, with regard to the AUC, is shown in bold.

**Table 4:** Description and possible values of the variables identified by the proposed decision tree approach.

**Factor**	**Description**	**Values**
**Age**	**Subject’s Age at Baseline**	**Numeric (Years)**
Single leg standing test with left leg	Single leg standing test with left leg. One leg standing test to measure the time the subject can stand on the left leg without support [55]. Best value in seconds of three tries.	Numeric (seconds)
Past smoking (cigarettes/day)	Cigarettes/day, on average, before quitting smoking.	Numeric
Diabetes Type 2	Medical history of the subject of diabetes Type 2	Yes No
Alcohol Consumption	Alcohol consumption frequency	Never Once a month or more rarely 2–4 times a month 2–3 times a week More than 4x a week
Number of Medications	Number of medications taken regularly by the subject	Numeric
Right hand strength	The subject’s hand strength, measured by the computerized dynamometer Grippit in an interval of 10 s, for the right hand.	Numeric (Newtons)
Left hand strength	The subject’s hand strength, measured by the computerized dynamometer Grippit in an interval of 10 s, for the left hand.	Numeric (Newtons)
Body mass index	Subject’s BMI	Numeric (kg/m^2^)
Backwards Digit Span Test score	The number of correct sequences on the Backwards digit span test [30].	Numeric
Present smoking frequency	Subject’s habit of smoking at baseline.	No, never smoked No, quit smoking Yes, smoke sometimes Yes, smoke regularly

## Supplementary Materials

The following are available online at https://www.mdpi.com/1660-4601/17/18/6674/s1, Table S1: Description of all input variables.
