# Supplementary material for: Multifactorial 10-Year Prior Diagnosis Prediction Model of Dementia
Source: Int J Environ Res Public Health. 2020 Sep 14;17(18):6674. doi: 10.3390/ijerph17186674 (PMC7557767; doi:10.3390/ijerph17186674)
Supplement: Supplementary file 1 [file ijerph-17-06674-s001.pdf]

Supplementary Material 1 – Description of all input variables

Table A1. List of the input variables with description, possible values and number of missing values

Variable Type	#	Variable	Description	Values	# Missing Values
Basic	1	Gender	Subject's gender	• Male<br>• Female	0
	2	Age	Subject's age	numeric	0
	3	BMI	Subject's BMI	numeric	0
Social	4	Education	Subject's highest level of education	• Unfinished elementary school<br>• Elementary school<br>• High school<br>• Vocational training<br>• University<br>• Doctorate	51 (5.2%)
	5	Religion	"Do you have a religious belief?"	• Yes<br>• No	57 (5.8%)
	6	ReligiousActivities	"Do you participate in religious activities?"	• Not at all<br>• Sometimes<br>• Often	39 (4%)
	7	VoluntaryAssociation	"If you are a member of a voluntary association, could you say that you feel strong associations with this association and its members?"	• Not a member of the association<br>• Highly<br>• To some extent<br>• Not especially<br>• Not at all	56 (5.7%)
	8	SocialNetwork	Assesses the subject's social network in terms of personal relationships and social interactions. See "Description 1"	• Very bad social network<br>• Bad social network<br>• Normal social network	57 (5.8%)

	9	SupportNetwork	Assesses the subject's support network in terms of people that can help them with life issues. See "Description 2"	numeric	53 (5.4%)
	10	Loneliness	Assesses the subject's feeling of loneliness. See "Description 3"	• Very high level of loneliness<br>• Some level of loneliness<br>• Normal level of loneliness	76 (7.8%)
Lifestyle	11	Exercise	Frequency of light exercise in last 12 months.	• Wheelchair or balance problems<br>• Never<br>• Once a month<br>• 2-3 times a month<br>• More than 3 times a month<br>• Every day	99 (10.1%)
	12	Alcohol_Consumption	"How often do you drink alcohol"	• Never<br>• Once a month or more rarely<br>• 2-4 times a month<br>• 2-3 times a week<br>• 4 or more times a week	46 (4.7%)
	13	Alcohol_Quantity	"How many "glasses" do you drink on a typical day when you drink alcohol?"	• Do not drink alcohol<br>• 1-2<br>• 3-4<br>• 5-6<br>• 7-9<br>• 10+	94 (9.6%)
	14	Working65	"When did you stop working?"	• Stopped working before 65 years	0

				• Worked until 65 years<br>• Still working/worked after 65 years	
	15	PresentSmoker	Do you smoke?	• Yes, smoke regularly<br>• Yes, smoke sometimes<br>• No, quitted smoking<br>• No, never smoked	34 (3.5%)
	16	PastSmoker_CigarsDay	If you have quitted smoking, how many cigarettes/day did you smoke on average before you stopped?	numeric	63 (6.4%)
	17	SocialActivities	Assesses the subject's engagement in sociocultural activities in the past 12 months. See "Description 4".	numeric	85 (8.6%)
	18	PhysicallyDemandingActivities	Assesses the subject's engagement in physically demanding activities in the past 12 months. See "Description 5".	numeric	85 (8.6%)
	19	LeisureActivities	Assesses the subject's engagement in leisure and hobby activities in the past 12 months. See "Description 6".	numeric	96 (9.8%)
Medical History	20	Medications	Number of medications taken regularly by the subject	numeric	0
	21	FamilyHistory	Subject's family (first degree relatives) medical history of importance, regarding cardiovascular disease, Parkinson's disease and dementia	• Yes<br>• No	2 (≈0%)
	22	Infarct	Subject's history of infarct	• Yes<br>• No	8 (≈0%)
	23	Arrhythmia	Subject's history of arrhythmia	• Yes<br>• No	13 (1.3%)

	24	HeartFailure	Subject's history of heart failure	• Yes<br>• No	1 (≈0%)
	25	Stroke	Subject's history of stroke	• Yes<br>• No	6 (≈0%)
	26	TIARIND	Subject's history of Transient Ischemic Attacks or Reversible Ischemic Neurological Deficit (TIA/RIND)	• Yes<br>• No	11 (1.1%)
	27	Diabetes_type1	Subject's history of diabetes type 1	• Yes<br>• No	1 (≈0%)
	28	Diabetes_type2	Subject's history of diabetes type 2	• Yes<br>• No	2 (≈0%)
	29	ThyroidDisease	Subject's history of thyroid disease	• Yes<br>• No	5 (≈0%)
	30	Cancer	Subject's history of cancer	• Yes<br>• No	1 (≈0%)
	31	Epilepsy	Subject's history of epilepsy	• Yes<br>• No	0
	32	AtrialFibrillation	Subject's history of atrial fibrillation	• Yes<br>• No	75 (7.7%)
	33	IschemicSigns	Subject's history of ischemic signs	• Yes<br>• No	76 (7.8%)
	34	Parkinsons	Subject's history of Parkinson's disease	• Yes<br>• No	3 (≈0%)
	35	Depression	Subject's history of depression	• Yes<br>• No	0
	36	OtherPsychiatricDiseases	Subject's history of other psychiatric diseases	• Yes<br>• No	5 (≈0%)
	37	Snoring	Subject's history of snoring	• Yes<br>• No	3 (≈0%)
	38	SleepApnea	Subject's history of sleep apnea	• Yes<br>• No	7 (≈0%)

	39	HipFracture	Subject’s history of hip fracture	• Yes<br>• No	10 (1.0%)
	40	HeadTrauma	Subject’s history of head trauma	• Yes<br>• No	8 (≈0%)
	41	DevelopmentalDisabilities	Subject’s history of developmental disabilities	• Yes<br>• No	0
	42	HighBloodPressure	Subject’s history of high blood pressure	• Yes<br>• No	11 (1.1%)
Blood Test	43	HB	Blood test analysis of the amount of haemoglobin in the blood (g/L)	numeric	19 (1.9%)
	44	CRP	Blood test analysis of the amount of C-reactive protein in the blood (mg/L)	numeric	30 (3.0%)
Physical Examination	45	Pain	“Have you had pain in the last 4 weeks?”	• Yes<br>• No	53 (5.4%)
	46	HeartRate_Sitting	Subject’s heart rate in beats per minute, while sitting	numeric	16 (1.6%)
	47	HeartRate_Lying	Subject’s heart rate in beats per minute, while lying	numeric	25 (2.5%)
	48	BloodPressure_Right	Subject’s systolic blood pressure measured on the right arm, while lying (mmHg).	numeric	21 (2.1%)
	49	HandStrength_Right	Subject’s right hand strength in Newtons, during an interval of 10s, measured by the Grippit instrument.	numeric	85 (8.6%)
	50	HandStrength_Left	Subject’s left hand strength in Newtons, during an interval of 10s, measured by the Grippit instrument.	numeric	88 (8.9%)
	51	Rise_Safe	“Does it feel "safe" for you to rise from a chair without using your arms?”	• Yes<br>• No, it feels unsafe<br>• Cannot stand up	63 (6.4%)
	52	Rise_How	Rising from the chair. How?	• Got up without using their arms	67 (6.8%)

				• Got up, but used their arms<br>• Tried but couldn't<br>• Not tried for security reasons<br>• Not tried as there was no suitable chair<br>• On a wheelchair	
	53	WeightLoss_3months	Any weight loss during the last 3 months?	• Yes, more than 3kg<br>• Don't know<br>• Yes, more than 1 kg, but less than 3 kg<br>• No weight loss	16 (1.6%)
	54	StandingTest_Right	Single leg standing with right leg. Best value in seconds of three tries.	numeric	72 (7.3%)
	55	StandingTest_Left	Single leg standing with left leg. Best value in seconds of three tries.	numeric	72 (7.3%)
	56	Dental_Prothesis	Assessment via x-ray of the subject's jaws in regards to their own teeth and prosthesis.	• Only own teeth<br>• Own teeth and removable dentures<br>• Own teeth as well as removable prosthesis in one tooth jaw, or toothless and whole prosthesis in one tooth jaw<br>• Completely toothless<br>• Completely toothless and complete denture in one or both jaws<br>• With implants	4 (≈0%)

	57	Dental_TeethNumber	Assessment via x-ray of the subject's jaws in regards to the number of own teeth.	numeric	96 (9.8%)
Psychological	58	MemoryLoss	Assessment of the subjects' memory in daily life situations. See "Description 7".	numeric	91 (9.3%)
	59	MemoryDecline	"Do you think your memory has gotten worse?"	• No<br>• Somewhat<br>• A lot	10 (1.0%)
	60	MemoryDecline2	"Does anyone in your circle think that your memory has gotten worse?"	• Yes<br>• No	18 (1.8%)
	61	AbstractThinking	"Explain the following phrase: 'The apple does not fall far from the tree'"	• Wrong answer<br>• Wrong, only concrete answer<br>• Wrong abstract answer<br>• Right answer	46 (4.7%)
	62	PersonalityChange	Assesses if the subject experienced changes regarding personality traits. See "Description 8"	• Yes<br>• No	19 (1.9%)
	63	Identity	First, the subject is asked questions about their identity: first name, last name, year of birth, date of birth and age. Then, the S_Psychological_Identity index is calculated as the sum of correct answers given by the subject.	numeric	6 (≈0%)
Health Instruments	64	SOC	Sense of Coherence [1]: assesses the subject's comprehensibility (how they perceive events as making logical sense), manageability (how they feel they can cope with situations), and meaningfulness (how they feel that life makes sense	numeric	56 (5.7%)

			and challenges are worthy overcomming).		
	65	DigitSpan_Forward	Forward Digit Span Test [2]: The tester say to the subject a sequence of numbers and the subject has to repeat it in the way they hear it. It starts with two 3-number sequence, going up to two 9-number sequence. Each correct sequence said by the subject counts as 1 point.	numeric	12 (1.2%)
	66	DigitSpan_Backwards	Backward Digit Span Test [2]: The tester say to the subject a sequence of numbers and the subject has to repeat it backwards. It starts with two 3-number sequence, going up to two 9-number sequence. Each correct sequence said by the subject counts as 1 point.	numeric	14 (1.4%)
	67	Livingston	Livingston Index [3]: a sleep disorder scale, composed of eight items regarding difficulty falling asleep or staying asleep, sleep medication usage, sleep interrupted at night, moods or tension, difficulty sleeping owing to pain or itching, inability to return to sleep after walking at night, waking up too early, or feeling tired more than two hours a day.	• No sleeping problems<br>• Presence of sleeping problems	31 (3.2%)
	68	EQ5D	EuroQol (EQ-5D) Index [4]: a generic instrument (non-disease specific) that aims to assess physical, mental and social functioning. The instrument is filled	• High quality of life<br>• Low quality of life	77 (7.9%)

			by the subject who describes their own health-related quality of life in regards to mobility, self-care, usual activities, pain/discomfort and anxiety/distress. We used a dichotomised version of EQ-5D by the lower quartile index values.		
	69	Index_Katz	Katz Index of Independence in Activities of Daily Living (ADL) [5]: Assesses functional status of the subject in regards to their capacity to perform activities of daily living independently.	• Severe impairment<br>• Moderate impairment<br>• Full function	17 (1.7%)
	70	IADL	Lawton Instrumental Activities of Daily Living (IADL) [6]: Assesses independent living skills of the subjects. Considers more complex skills than the ADL index.	• Dependent<br>• Independent	21 (2.1%)
	71	MMSE	Mini-Mental State Examination (MMSE) [7]: Assesses the cognitive aspects of mental functions.	numeric	5 (≈0%)
	72	ClockTest_Sum	Clock Drawing test [8]: Assesses cognitive impairment of a subject, in regards to verbal understanding, memory, spatially coded knowledge and construction skills. The 10-point score version was used in this study.	numeric	37 (3.7%)
	73	MCS12	Dichotomised Mental Composite Score of the SF-12 Health Survey [9]. The SF-12 is composed of 12 weighted questions that assess mental and physical functioning, and health-related quality of life. The Mental Composite Score is	• Low level of health<br>• High Level of health	98 (10.0%)

			calculated from the designated questions and compared to the age-specific mean. An age-specific mean difference score of -5.5 points indicates a low level of health.		
	74	PCS12	Dichotomised Physical Composite Score of the SF-12 Health Survey [9]. The SF-12 is composed of 12 weighted questions that assess mental and physical functioning, and health-related quality of life. The Physical Composite Score is calculated from the designated questions and compared to the age-specific mean. An age-specific mean difference score of -5.5 points indicates a low level of health.	• Low level of health<br>• High level of health	98 (10.0%)
	75	CPRS	Comprehensive Psychopathological Rating Scale [10]: It assesses the psychiatric state of the subject as to their level of depression.	• Absence of depression<br>• Mild depression<br>• Moderate depression<br>• Severe depression	13 (1.3%)

Description 1: SocialNetwork
This is a categorical index that assesses the subjects' social network into one of the following categories: "Very bad social network", "Bad social network" and "Normal social network". It is built upon the questions described below, whose alternatives are attributed to a value held in parenthesis. The sum score of the values determines the category, as the following: a sum score of 5 characterizes a "Very bad social network"; a sum score in the range of 1 to 4 characterizes a "Bad social network"; a sum score of 0 characterizes a "Normal social network".

Questions:

- Do you think your number of friends is enough?
  - Too few (1)
  - Enough (0)
  - Too many (0)

- How many people do you think you know well and can talk about most of the time?
  - No one (1)
  - 1-3 (1)
  - 4-6 (0)
  - 7-9 (0)
  - 10-15 (0)
  - 16-30 (0)
  - More than 30 (0)

- Do you have someone who you feel you can be yourself in, who accepts you with all your merits and flaws?
  - Yes, without a doubt (0)
  - Yes, probably (0)
  - No, probably not (1)
  - Not at all (1)

- Do you feel close to your family (other than your husband, spouse, partner and children)?
  - Missing relatives (1)
  - Highly (0)
  - To some extent (0)
  - Not especially (1)
  - Not at all (1)

Description 2: SupportNetwork
This index was built as the sum score of the questions' value regarding the subjects' social support network. The highest the score the worse social support network the subject has. The alternatives for the questions (with their respective values in parenthesis) are the following: Yes, without a doubt (1); Yes, probably (2); No, probably not (3); Not at all (4).

Questions:

- Can you get help from someone or someone in case of illness or other practical problems?
- Do you know someone or someone who can help you to write an official letter or appeal a government decision?
- Do you know that you have someone or someone who can provide you with proper personal support to cope with the stress and the problems of life?

Description 3 – Loneliness
This is a categorical index that assesses the subjects' felling of loneliness into one of the following categories: "Very high level of loneliness", "Some level of loneliness" and "Normal level of loneliness". It is built upon the questions described below, whose alternatives are attributed to a value held in parenthesis. The sum score of the values determines the category, as the following: a sum score of 4 characterizes a "Very bad social network"; a sum score in the range of 1 to 3 characterizes a "Some level of loneliness"; a sum score of 0 characterizes a "Normal level of loneliness".

Questions:

- Do you feel lonely?
  - Yes, often (1)
  - Yes, sometimes (0)
  - No, rarely (0)
  - No, never (0)

- When you look back on the last five years of your life, which of the following options best suits you?
  - I have not felt loneliness at any time in the past 5 years (0)
  - I have experienced occasional occasions with loneliness (0)
  - I have experienced recurrent periods of loneliness (1)
  - I have lived with a more or less constant feeling of loneliness (1)

- Do you feel a strong affinity with your local community?
  - Highly (0)
  - To some extent (0)

    - Not especially (1)
    - Not at all (1)

- Are you in a group of friends who have or do something in common?
    - Yes (0)
    - No (1)

Description 4: SocialActivities
The subjects were asked if they engaged in the following sociocultural activities in the past 12 months from the date they responded the questionnaire: “Cinema, theatre, or concert”; “Restaurant, café, or pub”; “Church or religious meetings”; and “Study circle or course of some kind”. The possible answers were “yes” or “no”. The “SocialActivities” index was built as the number of “yes” answers given by the subject.

Description 5: PhysicallyDemandingActivities
The subjects were asked if they engaged in the following physically demanding activities in the past 12 months from the date they responded the questionnaire: “Gardening”; “Taking walks outside”; “Picking berries or mushrooms”; “Hunting or fishing”; “Knit, weave or sew”; “Painting, drawing or sculpting”; “Home repairs”; and “Repairing cars or other mechanical equipment”. The possible answers were “yes” or “no”. The “PhysicallyDemandingActivities” index was built as the number of “yes” answers given by the subject.

Description 6: LeisureActivities
The subjects were asked if they engaged in the following leisure activities in the past 12 months from the date they responded the questionnaire: “Reading the newspaper”; “Reading magazines”; “Reading books”; “Watching television”; “Playing games or cards”; “Playing musical instruments”; “Listening to music”; and “Using the internet or playing computer games”. The possible answers were “yes” or “no”. The “LeisureActivities” index was built as the number of “yes” answers given by the subject.

Description 7: MemoryLoss
This index was built as the sum score of the questions’ value about the subjects’ memory in daily life situations. The highest the score means the highest memory decline of the subject. The alternatives for the questions (with their respective values in parenthesis) are the following: Never (1); Rarely (2); Sometimes (3); Often (4); Always (5).

Questions:

- Do you happen to come to the store and have forgotten what to trade?
- Do you have trouble remembering what happened the day before?
- Do you lose or place things?

- Do you find it hard to know where you are?
- Do you find it difficult to find the right home / at department?
- Do you find it difficult to find the store / post office?
- Do you find it difficult to find in a foreign environment?

Description 8: PersonalityChange
The tester asks the subject if they felt like they changed in regards to the items in the questions below. A positive answer to 2 or more items defines a change in personality.

Questions:
- More or less talkative?
- More or less grumpy?
- More or less agitated?
- More or less withdrawn?
- More or less apathetic?
- More or less worried?
- More difficult than before to make decisions?
- More difficult than before to take the initiative?

References
[1] Antonovsky, Aaron. "The structure and properties of the sense of coherence scale." Social science & medicine 36.6 (1993): 725-733. NBR 6023
[2] Wechsler, D. (1997). The Wechsler adult intelligence scale-III. San Antonio, TX: Psychological Corporation.
[3] Livingston, G., B. Blizard, and A. Mann. "Does sleep disturbance predict depression in elderly people? A study in inner London." Br J Gen Pract 43.376 (1993): 445-448. NBR 6023
[4] Brooks, Richard, and EuroQol Group. "EuroQol: the current state of play." Health policy 37.1 (1996): 53-72.
[5] Katz, Sidney. "Assessing self-maintenance: activities of daily living, mobility, and instrumental activities of daily living." Journal of the American Geriatrics Society 31.12 (1983): 721-727.
[6] Lawton, M. Powell, and Elaine M. Brody. "Assessment of older people: self-maintaining and instrumental activities of daily living." The gerontologist 9.3_Part_1 (1969): 179-186. NBR 6023
[7] Folstein, Marshal F., Susan E. Folstein, and Paul R. McHugh. "“Mini-mental state”: a practical method for grading the cognitive state of patients for the clinician." Journal of psychiatric research 12.3 (1975): 189-198. NBR 6023
[8] Agrell, Berit, and Ove Dehlin. "The clock-drawing test." Age and ageing 27.3 (1998): 399-403. NBR 6023

[9] Jenkinson, Crispin, and Richard Layte. "Development and testing of the UK SF-12." Journal of health services research & policy 2.1 (1997): 14-18.
[10] Montgomery, Stuart A., and M. A. R. I. E. Åsberg. "A new depression scale designed to be sensitive to change." The British journal of psychiatry 134.4 (1979): 382-389.
